# Cross-lag association between TyG-CVAI and frailty index: evidence from a natural population cohort in southwestern China

**DOI:** 10.3389/fpubh.2026.1892429

**Published:** 2026-09-14

**Authors:** Hao Wang, Shuming Ji, Yuqin Gan, Ruoyu Luo, Kaili Dai

**Affiliations:** 1 Department of Critical Care Medicine, West China School of Public Health and West China Fourth Hospital, Sichuan University, Chengdu, China; 2 Institute of Hospital Management, West China Hospital, Sichuan University, Chengdu, China; 3 Department of Science and Technology, Clinical Research Management Office, West China Hospital, Sichuan University, Chengdu, China

**Keywords:** Chinese visceral adiposity index, cross-lag model, frailty index, insulin resistance, triglyceride-to-glucose

## Abstract

**Background:**

Frailty is a major public health challenge among middle-aged and older adults, closely associated with metabolic dysregulation and visceral fat accumulation. The product of the triglyceride-glucose index and the Chinese Visceral Adiposity Index (TyG-CVAI) comprehensively reflects insulin resistance and visceral adiposity, yet its bidirectional temporal association with the frailty index remains unclear. This study aimed to examine this bidirectional association using cross-lagged panel models and characterize the predominant prospective temporal association.

**Methods:**

This study included 2,476 participants from the China Multi-Ethnic Cohort study who completed both the 2018 baseline survey and the 2021 follow-up survey and had complete data. The frailty index was constructed using the cumulative deficit model, based on 25 health-deficit items. A two-wave cross-lagged panel model was developed, with adjustment for age, sex, ethnicity, educational attainment, marital status, smoking status, and alcohol consumption. Model fit was evaluated using the comparative fit index (CFI), Tucker-Lewis index (TLI), root mean square error of approximation (RMSEA), and standardized root mean square residual (SRMR). The robustness of the findings was assessed through sensitivity analyses that excluded overlapping components. Additional cross-lagged analyses were performed using the TyG index and CVAI separately to verify the advantages of the composite indicator. Subgroup analyses and restricted cubic spline regression were used to explore population heterogeneity and nonlinear exposure-response relationships.

**Results:**

Baseline TyG-CVAI significantly predicted follow-up frailty index (standardized *β* = 0.165, *p* < 0.001), whereas the reverse path was considerably weaker (standardized *β* = 0.035, *p* < 0.001), indicating a predominant prospective temporal association. Sensitivity analyses excluding overlapping indicators yielded consistent results. Supplementary cross-lagged analyses using the TyG index and CVAI separately confirmed that the composite TyG-CVAI indicator exhibited a stronger prospective association with the frailty index than either individual component, and demonstrated superior model fit. Subgroup analyses revealed this association was significantly stronger among participants aged under 60, ethnic minorities, and those without hypertension or diabetes. Restricted cubic spline regression identified a significant non-linear relationship between baseline TyG-CVAI and follow-up frailty index, with an inflection point at approximately TyG-CVAI = 750.10 (the 50th percentile), beyond which the marginal association with frailty index attenuated.

**Conclusion:**

A significant bidirectional temporal association exists between TyG-CVAI and the frailty index, dominated by a positive prospective temporal association from TyG-CVAI to frailty, suggesting metabolic dysregulation may serve as a temporally preceding risk marker in frailty development. As a composite indicator based on routine clinical tests, TyG-CVAI may serve as a feasible biomarker for early frailty screening in community-dwelling middle-aged and older populations.

## 1 Introduction

Against the backdrop of rapid global population aging, frailty has emerged as a major public health challenge. It is highly prevalent among middle-aged and older adults and increases steeply with advancing age (1, 2). Clinical and epidemiological evidence has confirmed that frailty is closely linked to the onset and progression of multiple chronic conditions, and represents a key risk factor for metabolic diseases, cardiovascular diseases, chronic kidney disease, and type 2 diabetes mellitus in middle-aged and older populations (3–5). Moreover, frailty significantly elevates the risks of all-cause mortality (6), cardiovascular mortality, and physical disability, severely impairs health-related quality of life (7), and imposes a substantial disease burden on healthcare systems (8). Importantly, frailty is not an irreversible consequence of physiological aging; its progression can be effectively delayed or even reversed through early identification and targeted intervention (9). Therefore, early screening and risk stratification of frailty carry considerable public health and clinical value.

The pathogenesis of frailty is multifactorial, involving chronic low-grade inflammation, oxidative stress, metabolic dysregulation, and skeletal muscle loss. Among these pathways, metabolic disorders and visceral fat accumulation have been recognized as critical pathophysiological factors associated with frailty development and progression (10, 11). Visceral adipose tissue acts as an active endocrine organ rather than a passive energy depot; excessive visceral fat accumulation triggers systemic low-grade inflammation by secreting various adipokines, impairs vascular endothelial function, and disrupts insulin signaling, ultimately leading to insulin resistance (12–14). Insulin resistance not only reduces metabolic efficiency and glucose and fatty acid utilization but also directly inhibits skeletal muscle anabolism and accelerates muscle protein breakdown, thereby promoting sarcopenia, which is also a critical pathological substrate of frailty. Sarcopenia further compromises physical function and metabolic flexibility, forming a self-reinforcing vicious cycle of visceral fat accumulation, insulin resistance, sarcopenia, and frailty. In addition, metabolic disturbances associated with insulin resistance and visceral adiposity disrupt energy homeostasis and nutritional status, exacerbating fatigue and physical decline, which are hallmark features of frailty. Thus, clarifying the longitudinal patterns linking metabolic–adipose abnormalities to frailty and identifying reliable composite biomarkers is essential for early frailty screening (15).

Compared with traditional frailty assessment tools, the Frailty Index (FI) (16), based on the cumulative deficit model, quantifies frailty by calculating the proportion of accumulated health deficits across multiple dimensions, including diseases, physiological measurements, and self-reported symptoms (17). The FI provides a sensitive, comprehensive, and objective reflection of the dynamic progression of frailty, and its longitudinal changes are strongly associated with adverse clinical outcomes (18). Suitable for large-scale population-based cohort studies, the FI has become one of the most widely used instruments in frailty research, offering a robust framework for investigating longitudinal associations between frailty and metabolic indicators (19).

The Triglyceride Glucose (TyG) index is a well-validated, non-invasive surrogate marker of insulin resistance, calculated using fasting triglyceride and glucose levels; it is easily obtainable, highly reproducible, and suitable for large-scale epidemiological settings and primary care. Recent longitudinal studies have confirmed that elevated TyG index is prospectively associated with increased frailty risk and accelerated frailty progression (20–22). The Chinese Visceral Adiposity Index (CVAI) is an ethnicity-specific metric tailored to assess visceral adiposity in Chinese populations, integrating age, body mass index, waist circumference, triglycerides, and high-density lipoprotein cholesterol. CVAI outperforms traditional anthropometric indicators (e.g., BMI, waist circumference) in evaluating visceral fat accumulation and has been linked to adverse cardiometabolic outcomes and functional decline (23–25). A nationwide prospective cohort study showed that CVAI had a linear dose–response relationship with the risks of cardiovascular disease, heart disease, and stroke. Compared with other obesity-related indicators, including BMI, waist circumference, and the visceral adiposity index, CVAI demonstrated the best predictive performance (26). Despite these advances, existing research remains limited to investigating TyG or CVAI separately in relation to frailty or related diseases. To date, no study has explored the composite metabolic index TyG-CVAI, which combines insulin resistance and visceral adiposity, in association with frailty, nor have any studies employed cross-lagged panel models to characterize the bidirectional temporal relationships and longitudinal dynamics between TyG-CVAI and the FI. This critical evidence gap limits our understanding of the sequential links between integrated metabolic risk and frailty progression.

Using data from a large-scale natural population cohort in southwestern China, this study employed cross-lagged panel models to examine the bidirectional longitudinal and temporal associations between TyG-CVAI and the FI, and to characterize the predominant prospective temporal association. We further explored effect modification by age, ethnicity, and comorbidity status to characterize population heterogeneity in these relationships. This study aims to validate a clinically feasible composite metabolic biomarker for early frailty screening in Chinese community-dwelling populations, provide evidence for precision prevention strategies for frailty, and strengthen the longitudinal evidence base linking metabolic dysfunction to frailty. By characterizing the temporal relationships between TyG-CVAI and frailty, we seek to offer new insights into the potential metabolic pathways related to frailty and inform targeted public health interventions.

## 2 Methods

### 2.1 Study design and participants

This prospective cohort study was conducted based on the China Multi-Ethnic Cohort (CMEC) Study (27), a large-scale population-based prospective cohort targeting multi-ethnic natural populations in southwestern China. This study extracted and analyzed data from the 2018 baseline survey and the 2021 first follow-up subset survey of CMEC. According to the formally published CMEC cohort study protocol (28), the baseline phase of this cohort covered nearly 100,000 individuals from the general population in southwest China. However, owing to constraints related to follow-up personnel and financial resources, the operational complexity of face-to-face field surveys, and the costs associated with repeated biospecimen collection and testing, the cohort protocol prespecified that no less than 10% of baseline participants would be sampled to establish a prioritized follow-up subset for face-to-face follow-up, including standardized physical examinations and biospecimen collection. The study protocol was approved by the Medical Ethics Review Board of Sichuan University (approval numbers: K2016038, K2020022), and all participants provided written informed consent prior to data collection. The entire study was performed in strict accordance with the ethical principles of the Declaration of Helsinki.

Eligible participants were those who completed both the 2018 baseline survey and the 2021 first follow-up survey of the CMEC, with complete data for calculating the FI and TyG-CVAI, as well as complete information on key covariates including hypertension, chronic kidney disease (CKD) and diabetes. Participants were excluded if they were lost to follow-up, had missing data required for FI or TyG-CVAI calculation, or lacked key covariate information. A total of 99,556 participants were enrolled in the CMEC baseline survey, and 2,476 participants were finally included in the analysis after applying exclusion criteria, the detailed process of inclusion and exclusion is shown in Figure 1. To assess the representativeness of the included sample and potential selection bias, we compared the baseline demographic and clinical characteristics between the final included participants and the overall CMEC baseline population (Supplementary Table 1).

**Figure 1:**
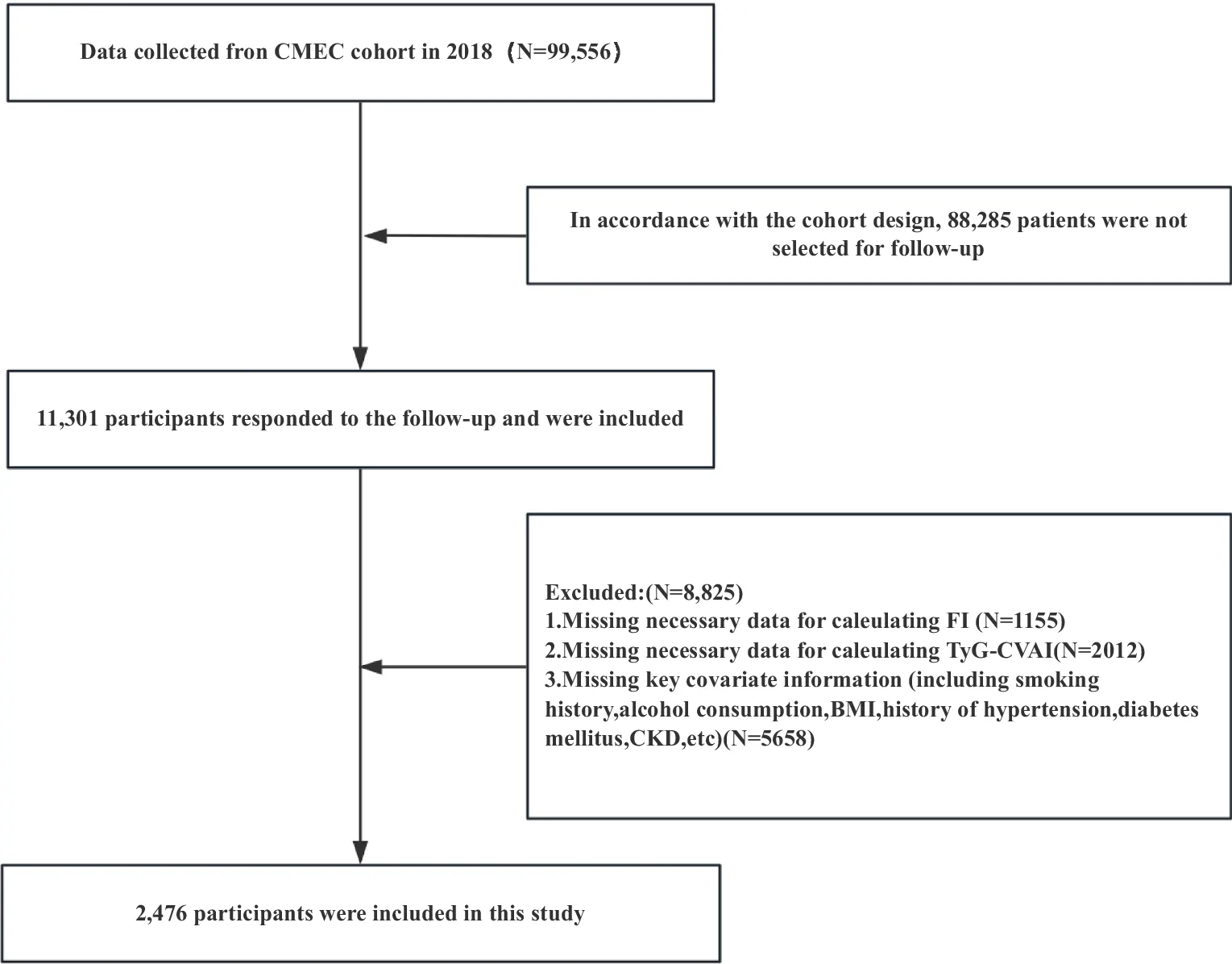
The study’s process of including and excluding the participants.

### 2.2 Data collection procedures

All data were collected following the standardized CMEC protocol with strict quality control to ensure accuracy, completeness and consistency. Data collection was divided into three standardized modules: questionnaire survey, physical examination and laboratory biochemical testing, all conducted by uniformly trained investigators.

#### 2.2.1 Standardized questionnaire survey

Face-to-face interviews were performed using a uniform electronic questionnaire by investigators who completed standardized training and passed qualification assessments. The questionnaire collected demographic characteristics (including age, sex, ethnicity, educational level, marital status, etc.), lifestyle factors (including smoking, alcohol consumption, etc.) and self-reported medical history (including diagnosis of chronic diseases, medication use, etc.). All survey processes were audio-recorded for quality inspection, and on-site data review was conducted daily to correct logical errors and missing items in a timely manner.

#### 2.2.2 Standardized physical examination

All physical indicators were measured following unified operating procedures. Height, weight, waist circumference (WC) and hip circumference were measured using calibrated instruments; body mass index (BMI) was calculated as weight (kg) divided by height squared (m^2^). Resting blood pressure and heart rate were measured three times consecutively with a standardized sphygmomanometer, and the average value was used for analysis. Peak expiratory flow (PEF) and ankle bone mineral density were tested with uniform professional equipment, and all measurements were completed by trained medical staff.

#### 2.2.3 Standardized laboratory biochemical test

Fasting venous blood samples (≥8 h of overnight fasting) were collected from all participants and tested at the designated central laboratory of the CMEC. All tests were performed in strict accordance with the National Clinical Laboratory Operation Procedures, including fasting blood glucose (GLU), triglycerides (TG), high-density lipoprotein cholesterol (HDL-C) and other metabolic indicators. Blood samples were processed and tested uniformly, with internal quality control implemented throughout the testing process to ensure the reliability of biochemical indicators.

### 2.3 Assessment of key variables

#### 2.3.1 Evaluation of frailty index

The FI was constructed using the cumulative deficit model, following the standardized FI construction procedure proposed by Searle et al. (17) and the optimized FI approach adapted for the Chinese population by Fan et al. (29). The criteria included the following: (1) deficits must be related to health status; (2) the prevalence of deficits must increase with age; (3) deficits must not reach saturation too early; (4) deficits should cover multiple organ systems; and (5) the same deficit items must be used at both time points. Considering the phenotypic characteristics of the multi-ethnic population in southwest China, 25 health-deficit indicators were ultimately selected for FI calculation. The complete list of all 25 health-deficit items, including their explicit definitions and coding rules, is provided in Supplementary Table 3. The FI was calculated using a ratio method, defined as the number of deficits present in each participant divided by the total number of deficit indicators (25 items). FI scores ranged from 0 to 1, with higher values indicating greater accumulation of health deficits and more severe frailty. According to widely accepted FI cutoffs, participants were classified into three mutually exclusive groups: robust (FI ≤ 0.1), prefrail (0.1 < FI < 0.25), and frail (FI ≥ 0.25). The same criteria were applied to calculate and classify FI at both baseline (2018) and the first follow-up (2021), thereby ensuring consistency in longitudinal assessment.

#### 2.3.2 Evaluation of TyG-CVAI

TyG-CVAI is a composite metabolic index that has been widely applied in several large-scale cohort studies for the comprehensive assessment of insulin resistance and visceral adiposity in Asian populations. It is calculated as the product of the TyG index and CVAI. A study based on the CHARLS cohort (30) demonstrated that elevated baseline TyG-CVAI was independently associated with incident cardiovascular disease (CVD) among middle-aged and older adults and showed better discriminatory performance than either TyG or CVAI alone. Consistent findings were reported by Fei et al. (31) in a cohort with a similar population profile. Wang et al. (32) systematically evaluated the longitudinal trajectories of TyG combined with various obesity-related indices and found that persistently high cumulative exposure to TyG-CVAI was significantly associated with an increased risk of CVD. Liu et al. (33) further reported that individuals with persistently high TyG-CVAI levels had the highest risk of CVD, while those whose exposure decreased from high to low levels remained at significantly elevated risk, suggesting that cumulative exposure to TyG-CVAI may be associated with persistent cardiovascular risk over time. All constituent indicators required for TyG and CVAI calculation were measured at both baseline and follow-up. The TyG Index is a well-established non-invasive surrogate marker for insulin resistance, calculated exclusively using TG and GLU levels (34). The formula is as follows:


$$\mathrm{TyG}\;\text{Index}=\ln(\frac{\begin{array}{l} \text{fasting triglyceride}\;(\mathrm{mg}/\mathrm{dl})\\ {}*\text{fasting glucose}\;(\mathrm{mg}/\mathrm{dl}) \end{array}}{2})$$

Fasting venous blood samples were collected after an overnight fast of at least 8 h, and TG and GLU concentrations were detected using a fully automatic biochemical analyzer with standardized internal quality control protocols throughout the testing process.

CVAI is an ethnicity-specific adiposity assessment index developed specifically for the Chinese population, which can precisely quantify the degree of visceral fat accumulation in Asian individuals. Gender-specific calculation formulas were applied, incorporating age, BMI, WC, TG, and HDL-C. The formulas for CVAI are presented below:

Calculating equation for male:


$$\begin{array}{l} \mathrm{CVA}I_{\text{male}}=-267.93+0.68*\mathrm{age}+0.03*\mathrm{BMI}(\mathrm{kg}/m^{2})\\ +4.00*\mathrm{WC}(\mathrm{cm})+22.00*\log(\mathrm{TG}(\mathrm{mg}/\mathrm{dL})*0.0113)\\ -16.32*\text{HDLC}(\mathrm{mg}/\mathrm{dL})*0.0259 \end{array}$$

Calculating equation for female:


$$\begin{array}{l} \mathrm{CVA}I_{\text{female}}=-187.32+1.71*\mathrm{age}+4.23\\ {}*\mathrm{BMI}(\mathrm{kg}/m^{2})+1.12*\mathrm{WC}(\mathrm{cm})+39.76\\ {}*\log(\mathrm{TG}(\mathrm{mg}/\mathrm{dL})*0.0113)-11.66\\ {}*\text{HDLC}(\mathrm{mg}/\mathrm{dL})*0.0259 \end{array}$$

The final TyG-CVAI composite index was derived by multiplying the TyG Index by the CVAI, using the following equation:


TyG−CVAI=TyGIndex∗CVAI

A higher TyG-CVAI value signifies a higher combined metabolic risk of insulin resistance and visceral fat accumulation in the study participants.

#### 2.3.3 Covariate assessment

Covariates were selected based on previous epidemiological evidence linking metabolic status to frailty and the analytical framework of this study. All covariates were ascertained through the standardized CMEC questionnaire, combined with objective physical examinations, laboratory biochemical tests, and cross-verification with medical records to ensure data accuracy and reliability. Covariates were classified into three core domains: (1) Demographic characteristics: age, sex, ethnicity, educational level, and marital status; (2) Lifestyle factors: smoking status and alcohol consumption; (3) Health status indicators: hypertension, chronic kidney disease (CKD), and diabetes mellitus.

For chronic disease-related covariates, participants were required to provide detailed information including the date of initial diagnosis, the hospital where the diagnosis was confirmed, and history of hospitalization. Investigators further validated the authenticity of disease information by cross-checking official medical records to minimize recall bias and misclassification.

### 2.4 Statistical analysis

All statistical analyses were performed using R software (version 4.5.1), with a two-sided test level of α = 0.05. Baseline characteristics were described according to FI classification (robust, pre-frail, frail). Normally distributed continuous variables were presented as mean ± standard deviation (SD) and compared using one-way analysis of variance (ANOVA); non-normally distributed continuous variables were presented as median (interquartile range) and compared using the Kruskal-Wallis H test. Categorical variables were presented as frequency (percentage) and compared using the chi-square test or Fisher’s exact test (if theoretical frequency <5). To evaluate selection bias, baseline characteristics were compared between the final included sample and the overall CMEC baseline population.

A two-time-point cross-lagged panel model (baseline and follow-up) was constructed as the primary analysis to investigate the longitudinal bidirectional temporal relationship and the predominant prospective temporal association between TyG-CVAI and FI. In the model, TyG-CVAI and FI at baseline were set as exogenous variables, TyG-CVAI and FI at follow-up period as endogenous variables, with adjustment for age, sex, ethnicity, educational level, marital status, smoking and alcohol consumption. The model included synchronous correlations of TyG-CVAI and FI at baseline and follow-up, as well as autoregressive paths of each variable from T0 to T1, to quantify the relative strength of the bidirectional temporal associations. Model fit was evaluated using the comparative fit index (CFI), Tucker-Lewis index (TLI), root mean square error of approximation (RMSEA), standardized root mean square residual (SRMR), Akaike information criterion (AIC), and Bayesian information criterion (BIC).

To assess the robustness of the primary findings and address the overlap in constituent components between the FI and TyG-CVAI, we performed four sensitivity analyses: (1) reconstructing the FI after excluding the BMI-related item; (2) reconstructing the FI after excluding the BMI- and diabetes-related items; (3) reconstructing the FI after excluding the BMI-, diabetes-, and CKD-related items; and (4) reconstructing the FI after additionally excluding the waist-to-hip ratio (WHR) item, together with the BMI-, diabetes-, and CKD-related items. Cross-lagged models were rebuilt for each modified FI, and changes in path regression coefficients were compared to confirm the stability of the association. In addition, to verify the advantages of the composite TyG-CVAI indicator over its individual components, supplementary cross-lagged panel models were constructed using the TyG index and CVAI separately in relation to the FI. Model fit indices and path coefficients were compared across the composite and individual indicator models.

Subgroup analyses were conducted to explore population heterogeneity in the association between TyG-CVAI and FI. Stratification factors included demographic characteristics (age <60 or ≥60 years, sex, ethnicity, educational level, marital status), lifestyle factors (smoking, alcohol consumption) and health status (hypertension, CKD, diabetes). A cross-lagged model was constructed for each subgroup, and between-group differences in the regression coefficients of the TyG-CVAI-to-FI path were tested.

Furthermore, restricted cubic spline regression models were used to explore the non-linear exposure-response relationships: (1) baseline FI as the independent variable and follow-up TyG-CVAI as the dependent variable; (2) baseline TyG-CVAI as the independent variable and follow-up FI as the dependent variable. The optimal number of knots was determined by comparing models with 3, 4, 5, and 6 knots using AIC and BIC, with the model yielding the smallest AIC and BIC values selected. Knots were placed at the 10th, 50th, and 90th percentiles of the exposure variable distribution.

## 3 Results

Among the 99,556 participants enrolled in the 2018 baseline survey of CMEC, 11,301 were selected for follow-up in 2021 and were considered for inclusion in the present study. After excluding 1,155 participants with missing FI data, 2,012 with missing TyG-CVAI data, and 5,658 with missing CKD data, a final analytic sample of 2,476 participants who completed both the baseline survey and the first follow-up survey in 2021 was obtained. The detailed inclusion and exclusion process, including the number of participants excluded at each step, is shown in Figure 1. To evaluate the representativeness of the included sample and potential selection bias, we compared the baseline demographic and clinical characteristics between the 2,476 included participants and the entire 99,556 CMEC baseline population, as presented in Supplementary Table 1. The included participants were significantly younger (48.85 ± 11.66 vs. 51.43 ± 11.80 years, *p* < 0.001), had a higher proportion of Han ethnicity (71.6% vs. 44.4%, *p* < 0.001) and a higher rate of education attainment at or above high school (31.1% vs. 22.0%, *p* < 0.001), and exhibited lower prevalence rates of hypertension (26.5% vs. 32.2%, *p* < 0.001); no significant between-group differences were observed in smoking history, alcohol consumption, CKD or diabetes prevalence (all *p* > 0.05).

The baseline characteristics of the included participants stratified by frailty status are summarized in Table 1, where participants were divided into robust (*n* = 634), pre-frail (*n* = 1,590), and frail (*n* = 252) groups according to the baseline FI value. Compared with the robust group, participants in the frail group were significantly older (58.50 ± 9.78 vs. 43.37 ± 9.77 years, *p* < 0.001), more likely to be male (64.3% vs. 53.0%, *p* = 0.008), belong to ethnic minority groups (46.4% vs. 11.2%, *p* < 0.001), have an education level below high school (89.3% vs. 46.7%, *p* < 0.001), be unmarried (18.3% vs. 6.9%, *p* < 0.001), a lower rate of smoking history (14.3% vs. 22.1%, *p* = 0.026), and consume alcohol less frequently (27.8% vs. 53.0%, *p* < 0.001). Notably, the prevalence rates of hypertension (71.4% vs. 3.6%), CKD (21.4% vs. 6.9%), and diabetes (34.5% vs. 1.3%) were markedly higher in the frail group than in the robust group (all *p* < 0.001), and both baseline and follow-up TyG-CVAI values showed a gradual upward trend from the robust to the pre-frail and frail groups (all *p* < 0.001).

**Table 1:** Description of the basic characteristics of the CMEC cohort population stratified by frailty index.

Variables	Overall (*N* = 2,476)	Grouping of the FI scores			*p* values
		Frail (*n* = 252)	Prefrail (*n* = 1,590)	Robust (*n* = 634)	
Age (years)	48.85 ± 11.66	58.50 ± 9.78	49.50 ± 11.47	43.37 ± 9.77	< 0.001
Age level					< 0.001
< 60 years old	1,958 (79.1%)	131 (52.0%)	1,251 (78.7%)	576 (90.9%)	
≥ 60 years old	518 (20.9%)	121 (48.0%)	339 (21.3%)	58 (9.1%)	
Sex					0.008
Male	1,411 (57.0%)	162 (64.3%)	913 (57.4%)	336 (53.0%)	
Female	1,065 (43.0%)	90 (35.7%)	677 (42.6%)	298 (47.0%)	
Ethnicity					< 0.001
Han	1,772 (71.6%)	135 (53.6%)	1,074 (67.5%)	563 (88.8%)	
Minority	704 (28.4%)	117 (46.4%)	516 (32.5%)	71 (11.2%)	
Educational level					< 0.001
< High school degree	1,707 (68.9%)	225 (89.3%)	1,186 (74.6%)	296 (46.7%)	
≥ High school degree	769 (31.1%)	27 (10.7%)	404 (25.4%)	338 (53.3%)	
Marital status					< 0.001
Married	2,222 (89.7%)	206 (81.7%)	1,426 (89.7%)	590 (93.1%)	
Divorced or single	254 (10.3%)	46 (18.3%)	164 (10.3%)	44 (6.9%)	
Smoking history	513 (20.7%)	36 (14.3%)	337 (21.2%)	140 (22.1%)	0.026
Alcohol consumption	1,044 (42.2%)	70 (27.8%)	638 (40.1%)	336 (53.0%)	< 0.001
Hypertension	655 (26.5%)	180 (71.4%)	452 (28.4%)	23 (3.6%)	< 0.001
Chronic kidney diseases	306 (12.4%)	54 (21.4%)	208 (13.1%)	44 (6.9%)	< 0.001
Diabetes	255 (10.3%)	87 (34.5%)	160 (10.1%)	8 (1.3%)	< 0.001
Baseline FI (actual score)	3.81 ± 1.83	7.45 ± 1.03	4.06 ± 1.01	1.74 ± 0.56	< 0.001
Baseline FI (standardized)	0.15 ± 0.07	0.30 ± 0.04	0.16 ± 0.04	0.07 ± 0.02	< 0.001
Baseline TyG-CVAI	−0.02 ± 0.98	0.84 ± 0.93	0.07 ± 0.95	−0.58 ± 0.76	< 0.001
Follow-up FI (actual score)	3.93 ± 1.83	6.62 ± 1.62	4.12 ± 1.49	2.40 ± 1.09	< 0.001
Follow-up FI (standardized)	0.16 ± 0.07	0.26 ± 0.06	0.16 ± 0.06	0.10 ± 0.04	< 0.001
Follow-up TyG-CVAI	−0.14 ± 0.99	0.63 ± 0.90	−0.07 ± 0.96	−0.64 ± 0.81	< 0.001

FI, frailty index; TyG-CVAI, Insulin resistance-visceral fat complex index.

A cross-lagged panel model adjusted for age, sex, ethnicity, educational level, marital status, smoking, and alcohol consumption was constructed to investigate the longitudinal bidirectional association between TyG-CVAI and FI, with the results displayed in Figure 2. The model fit indices for the primary cross-lagged model and all sensitivity verification models are presented in Table 2. Significant autoregressive paths were identified for both TyG-CVAI (standardized *β* = 0.742, *p* < 0.001) and FI (standardized *β* = 0.617, *p* < 0.001) from baseline to follow-up, confirming the favorable temporal stability of these two indicators over the 3-year follow-up period. For the cross-lagged paths, baseline TyG-CVAI was significantly and positively associated with follow-up FI (standardized *β* = 0.165, *p* < 0.001), whereas the association of baseline FI with follow-up TyG-CVAI was considerably weaker (standardized *β* = 0.035, *p* < 0.001), indicating a predominant prospective temporal association from TyG-CVAI to FI rather than the reverse cross-lag pathway.

**Figure 2:**
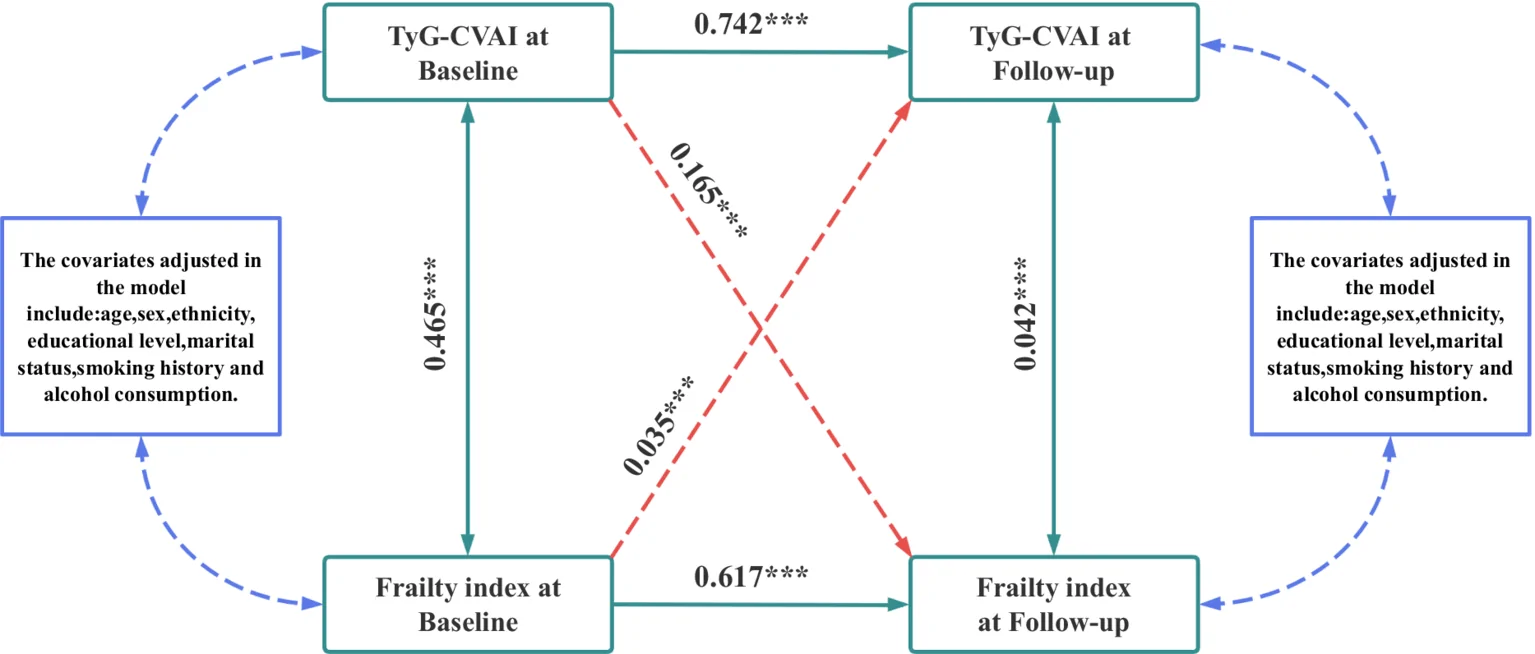
The cross-lagged effects path diagram between FI and TyG-CVAI at the baseline and follow-up time points, in participants from the CMEC. The path coefficients all have been standardized, and the curved bidirectional arrows represent the covariates considered by the model.

**Table 2:** The fitting indicators of the main cross-lagged model and each sensitivity verification model.

Model	RMSEA	95% CI of RMSEA		CFI	TLI	SRMR	AIC	BIC
		Lower	Upper					
FI and TyG-CVAI	0.151	0.146	0.156	0.881	0.711	0.097	22402.782	22565.585
FI and TyG-CVAI (FI excluded BMI)	0.172	0.163	0.181	0.835	0.599	0.103	22935.953	23098.734
FI and TyG-CVAI (FI excluded BMI and diabetes)	0.174	0.165	0.183	0.825	0.575	0.103	23180.527	23343.307
FI and TyG-CVAI (FI excluded BMI, diabetes and CKD)	0.174	0.165	0.183	0.825	0.575	0.103	23182.403	23345.184
FI and TyG	0.215	0.204	0.225	0.782	0.434	0.123	24033.654	24173.199
FI and CVAI	0.276	0.263	0.290	0.840	0.521	0.187	22307.748	22447.293

RMSEA, root mean square error of approximation; CFI, comparative fit index; TLI, Tucker-Lewis index; SRMR, standardized root mean square residual; AIC, akaike information criterion; BIC, bayesian information criterion.

To assess the robustness of the primary findings and address potential overlap in constituent components between the FI and TyG-CVAI, four sensitivity analyses were conducted by reconstructing the FI after excluding the overlapping indicators. The results are presented in Supplementary Figures 1–4. After excluding BMI-related components from the FI, the prospective temporal association between baseline TyG-CVAI and follow-up FI remained statistically significant (standardized *β* = 0.133, *p* < 0.001), while the reverse path was weakened (standardized *β* = 0.015, *p* < 0.05); when both BMI-related and diabetes-related components were removed from the FI, the forward path from TyG-CVAI to FI was still significant (standardized *β* = 0.117, *p* < 0.001) and the reverse path became non-significant (standardized *β* = 0.011, *p* > 0.05); furthermore, after excluding BMI-related, diabetes-related, and CKD-related components from the FI, the results remained highly consistent, with baseline TyG-CVAI remaining significantly associated with higher follow-up FI (standardized *β* = 0.117, *p* < 0.001) and no significant reverse association observed (standardized *β* = 0.011, *p* > 0.05). In addition, we conducted a fourth sensitivity analysis by further excluding the WHR item from the FI. These sensitivity analyses consistently confirmed that the cross-lagged association from TyG-CVAI to FI was stable and independent of the overlapping variables between the two indicators.

To verify the advantages of the composite TyG-CVAI indicator over its individual components, supplementary cross-lagged panel models were constructed using the TyG index and CVAI separately in relation to the FI, with results presented in Supplementary Figures 5, 6 and model fit indices summarized in Table 2. In the TyG-only model, the standardized path coefficient from baseline TyG to follow-up FI was 0.066, which was substantially smaller than the corresponding coefficient of 0.165 in the composite TyG-CVAI model. In the CVAI-only model, the reverse path coefficient from baseline FI to follow-up CVAI was 0.022, which was also smaller than the corresponding coefficient of 0.035 in the composite TyG-CVAI model and did not reach statistical significance. Collectively, these findings indicate that the composite TyG-CVAI index demonstrates a stronger prospective temporal association with FI and better model fit.

Subgroup analyses stratified by demographic characteristics, lifestyle factors, and chronic disease status were performed to explore the potential effect modification on the bidirectional cross-lagged paths between TyG-CVAI and FI, with detailed results presented in Table 2. For the path from baseline FI to follow-up TyG-CVAI, this association remained statistically significant in most subgroups, with only the subgroups of participants with smoking history and those with diabetes showing non-significant results as their 95% confidence intervals crossed zero; notably, the *p*-values for interaction across all stratification variables were non-significant, indicating that the strength of this path was not significantly modified by any of the included grouping factors. In contrast, the path from baseline TyG-CVAI to follow-up FI was consistently positive and statistically significant across all subgroups (all *p* < 0.05), and significant effect modifications were detected for age, ethnicity, hypertension status, and diabetes status. Specifically, the effect size of this path was significantly stronger in participants aged <60 years (standardized *β* = 0.204, 95%CI: 0.187–0.222) than in those aged ≥60 years (standardized *β* = 0.116, 95%CI: 0.081–0.150); stronger in ethnic minority participants (standardized *β* = 0.195, 95%CI: 0.173–0.217) than in Han participants (standardized *β* = 0.155, 95%CI: 0.132–0.178); stronger in participants without hypertension (standardized *β* = 0.197, 95%CI: 0.177–0.216) than in those with hypertension (standardized *β* = 0.117, 95%CI: 0.090–0.145); and stronger in participants without diabetes (standardized *β* = 0.182, 95%CI: 0.165–0.199) than in those with diabetes (standardized *β* = 0.090, 95%CI: 0.044–0.136). No significant effect modification was observed for sex, educational level, marital status, smoking history, alcohol consumption, or CKD status for this path (all *p*-values for interaction >0.05) (see Table 3).

**Table 3:** The cross-lagged associations between FI and TyG-CVAI in each subgroup analysis.

Variables	Baseline FI to follow-up TyG-CVAI		Baseline TyG-CVAI to follow-up FI	
	Standardized coefficient with 95% CI	*p* values	Standardized coefficient with 95% CI	*p* values
Age level		0.814		< 0.001
< 60 years old	0.055 (0.038–0.072)		0.204 (0.187–0.222)	
≥ 60 years old	0.051 (0.025–0.077)		0.116 (0.081–0.150)	
Sex		0.819		0.191
Male	0.050 (0.025–0.074)		0.168 (0.142–0.193)	
Female	0.053 (0.036–0.070)		0.190 (0.169–0.211)	
Ethnicity		0.827		0.013
Han	0.057 (0.037–0.076)		0.155 (0.132–0.178)	
Minority	0.054 (0.033–0.074)		0.195 (0.173–0.217)	
Educational level		0.230		0.461
< High school degree	0.050 (0.035–0.066)		0.172 (0.154–0.190)	
≥ High school degree	0.073 (0.040–0.106)		0.186 (0.155–0.216)	
Marital status		0.363		0.090
Married	0.059 (0.043–0.074)		0.183 (0.167–0.200)	
Divorced or single	0.039 (0.001–0.078)		0.138 (0.088–0.188)	
Smoking history		0.238		0.679
No	0.059 (0.044–0.075)		0.179 (0.162–0.197)	
Yes	0.034 (−0.005–0.073)		0.171 (0.134–0.208)	
Alcohol consumption		0.180		0.480
No	0.049 (0.031–0.066)		0.184 (0.162–0.205)	
Yes	0.069 (0.045–0.093)		0.172 (0.148–0.196)	
Hypertension		0.882		< 0.001
No	0.041 (0.022–0.060)		0.197 (0.177–0.216)	
Yes	0.038 (0.012–0.064)		0.117 (0.090–0.145)	
Chronic kidney diseases		0.417		0.518
No	0.055 (0.028–0.083)		0.210 (0.179–0.240)	
Yes	0.083 (0.022–0.143)		0.177 (0.081–0.272)	
Diabetes		0.852		< 0.001
No	0.051 (0.036–0.066)		0.182 (0.165–0.199)	
Yes	0.046 (−0.004–0.096)		0.090 (0.044–0.136)	

FI, frailty index; TyG-CVAI, Insulin resistance-visceral fat complex index.

Restricted cubic spline regression models were applied to explore the non-linear exposure-response relationships between TyG-CVAI and FI, as illustrated in Figures 3, 4. The optimal number of knots was determined by comparing models with 3, 4, 5, and 6 knots using AIC and BIC. For both the “baseline FI to follow-up TyG-CVAI” and “baseline TyG-CVAI to follow-up FI” models, the 3-knot model yielded the smallest AIC and BIC values (Supplementary Table 2), and was therefore selected as the final model. The three knots were placed at the 10th, 50th, and 90th percentiles of the exposure variable distribution. For baseline TyG-CVAI, the corresponding knot values were 207.23, 750.10, and 1395.35; for baseline FI, the corresponding knot values were 1.60, 3.60, and 6.30. For the association between baseline FI and follow-up TyG-CVAI, a significant linear positive trend was identified (*P* for overall trend = 0.016), with no significant non-linear component (*P* for nonlinearity = 0.694). For the association between baseline TyG-CVAI and follow-up FI, a significant positive non-linear relationship was observed (*P* for overall trend<0.001, *P* for nonlinearity = 0.003), with follow-up FI increasing as the baseline TyG-CVAI rose, and exhibited a nonlinear characteristic of rapid increase in the early stage and a slower increase in the later stage. The inflection point of the exposure-response curve was located at approximately TyG-CVAI = 750.10 (corresponding to the 50th percentile of the study population). Specifically, when baseline TyG-CVAI was below 750.10, follow-up FI increased at a relatively rapid rate with rising TyG-CVAI; when TyG-CVAI exceeded this threshold, the rate of FI increase attenuated markedly, exhibiting a pattern of diminishing marginal association. This further supports the prospective temporal association between TyG-CVAI and frailty progression.

**Figure 3:**
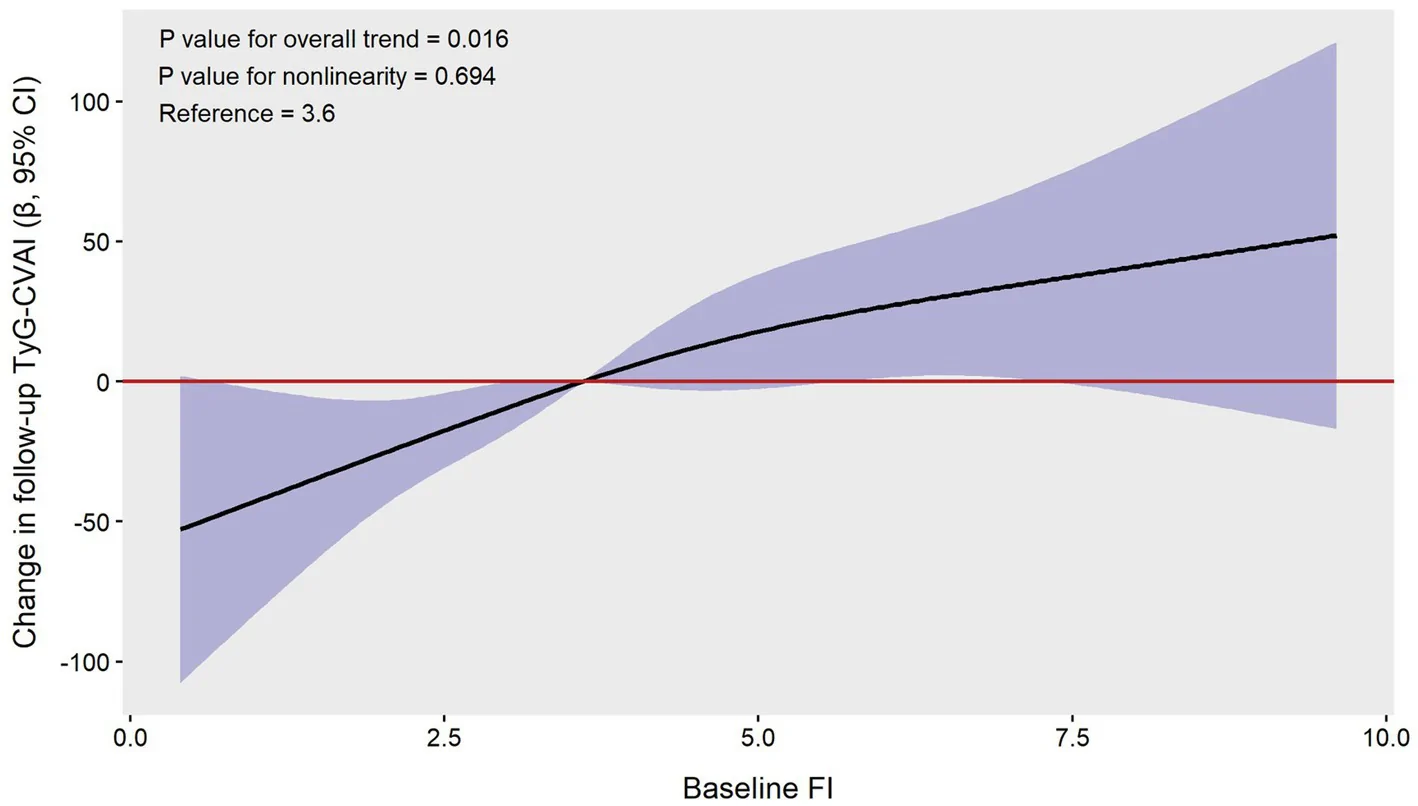
The nonlinear exposure-response association graph between baseline FI and follow-up TyG-CVAI.

**Figure 4:**
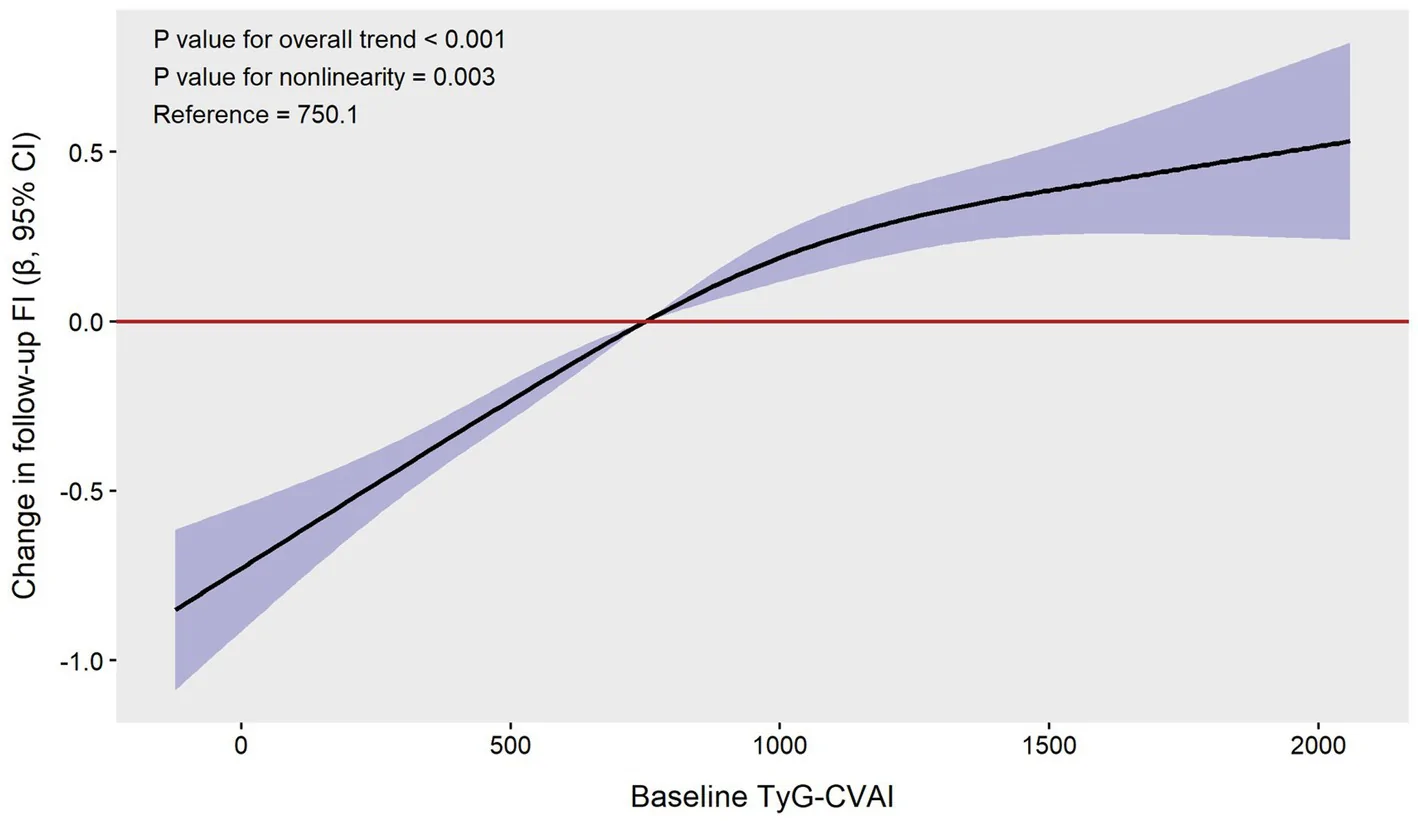
The nonlinear exposure-response association graph between baseline TyG-CVAI and follow-up FI.

## 4 Discussion

This prospective cohort study based on the CMEC in southwestern China, adopted cross-lagged panel models, subgroup analyses and restricted cubic spline regression to explore the temporal prospective associations between the TyG-CVAI and the FI at baseline and follow-up. Our findings revealed an asymmetric temporal association between TyG-CVAI and FI: a stronger positive prospective association was observed from baseline TyG-CVAI to follow-up FI, whereas the association from baseline FI to follow-up TyG-CVAI was comparatively weaker. These primary results remained consistent in a series of sensitivity analyses that excluded overlapping components between FI and TyG-CVAI, supporting the stability of this temporal trend. Furthermore, significant effect modification was detected in the prospective association of baseline TyG-CVAI with follow-up FI across age, ethnicity, hypertension and diabetes subgroups, while non-linear exposure-response patterns further characterized the gradient relationship between TyG-CVAI and frailty progression.

The baseline characteristics of the study population were consistent with established epidemiological patterns of frailty among middle-aged and older Chinese adults. The overall mean baseline FI was 0.15 ± 0.07, with 10.2% classified as frail, 64.2% as pre-frail, and 25.6% as robust. This distribution is similar to the epidemiological features of frailty in middle-aged and older adults reported by the China Health and Retirement Longitudinal Study (CHARLS) (35), but lower than the prevalence of frailty in European and American populations (approximately 24%) (2), which may be attributed to the younger mean age of the sample in this study (48.85 years). As an age-related syndrome, frailty has not yet reached a high incidence stage in young and middle-aged adults (36). In this study, the mean age of frail participants (58.50 years) was significantly higher than that of robust participants (43.37 years), which further confirmed the impact of age on frailty. Regarding the distribution of TyG-CVAI, both baseline and follow-up TyG-CVAI levels in the frail group were significantly higher than those in the pre-frail and robust groups. The follow-up TyG-CVAI level was slightly higher than the baseline level in the overall population, suggesting that the combined metabolic risk of visceral fat accumulation and insulin resistance in populations from southwestern China showed a slow upward trend over time. This finding is consistent with the globally increasing prevalence of metabolic syndrome, and provides population-level contextual support for the association between metabolic disorders and frailty.

The cross-lagged model in the present study showed that the prospective temporal association between TyG-CVAI and FI was substantially stronger than the reverse pathway, suggesting that metabolic dysfunction may temporally precede frailty progression and serve as a potential risk marker. It should be noted that cross-lagged panel analysis can establish temporal ordering but cannot confirm causality. The observed directional predominance provides evidence of temporal precedence; however, it cannot exclude the possibility of reverse causation resulting from unmeasured confounding factors or other pathways not captured by the model. This finding is consistent with mainstream studies on the association between metabolic syndrome and frailty, which have reported that metabolic abnormalities may accelerate the progression of frailty through multiple biological pathways (37, 38). From a mechanistic perspective, the observation that TyG-CVAI temporally preceded frailty is supported by multiple lines of evidence. A recent Mendelian randomization analysis reported evidence supporting a potential link between a higher TyG index and an increased risk of frailty (*β* = 0.214, 95% CI: 0.079–0.349; *p* = 0.002), providing genetic evidence that insulin resistance may be linked to frailty development (39). A multi-cohort analysis based on four large prospective cohorts (CHARLS, HRS, SHARE, and MHAS) further demonstrated a dose–response relationship between metabolic dysregulation and frailty, showing that each additional metabolic abnormality component (central obesity, hypertension, or hyperglycemia) may synergistically accelerate frailty progression (40). In addition, a nationwide prospective cohort study showed that the TyG index was independently associated with abnormal blood pressure subtypes in older adults; each 1-unit increase in the TyG index was associated with a 39% higher risk of hypertension (OR = 1.39, 95% CI: 1.18–1.63), supporting the link between insulin resistance and cardiometabolic dysfunction, a process that may contribute to frailty progression (41). Furthermore, previous evidence has shown a dose–response relationship between CVAI and the risk of cardiovascular disease; each 1-standard-deviation increase in CVAI was associated with a 17% higher risk of CVD, a 12% higher risk of heart disease, and a 31% higher risk of stroke (26). These cardiovascular consequences of visceral adiposity accumulation may represent potential pathways linking higher TyG-CVAI to frailty progression. Notably, the supplementary cross-lagged analyses using the TyG index and CVAI separately further demonstrated the superiority of the composite indicator TyG-CVAI. This apparent advantage may partly reflect the complementary pathophysiological information captured by the two components. The TyG index primarily reflects systemic insulin resistance and disturbances in glucose and lipid metabolism (39), whereas CVAI is designed to quantify visceral adiposity (42). Visceral adipose tissue, as a metabolically active endocrine organ, may contribute to chronic low-grade inflammation and adipokine dysregulation (43). However, triglycerides are incorporated into both the TyG index and the CVAI formula, indicating that the composite TyG-CVAI index is not derived from two entirely independent physiological signals and is mathematically weighted toward triglycerides. Consequently, part of the apparent advantage of TyG-CVAI over its individual components may reflect mathematical redundancy arising from the shared triglyceride component, rather than exclusively representing independent and complementary biological information (44). By integrating these metabolic and adiposity-related dimensions, TyG-CVAI may provide a more comprehensive characterization of the metabolic–adiposity axis relevant to frailty development; however, the extent to which its apparent advantage reflects genuinely complementary information rather than shared variance remains to be further clarified.

In addition, the temporal stability of TyG-CVAI as a composite index was significantly higher than that of FI, reflecting the relatively persistent nature of insulin resistance and visceral fat accumulation. Sensitivity analyses further confirmed that this temporal pattern remained unchanged after excluding FI components overlapping with TyG-CVAI (including BMI-related, diabetes-related and chronic kidney disease-related items), indicating that the observed association was not driven by shared indicator composition and was robust to model specification. As a comprehensive index reflecting multi-system functional reserve, FI is susceptible to short-term disturbances such as acute illnesses and psychological stress. This difference in indicator properties further supports the stable prospective association of TyG-CVAI with FI.

Subgroup analyses revealed population heterogeneity in the prospective associations, which provides important clues for targeted risk screening. The prospective association of TyG-CVAI with FI was significantly stronger in adults under 60 years old than in those aged 60 years and older. This result appears to contradict the common perception that frailty is more prevalent in older adults, but actually reflects the advantages of metabolic plasticity in young and middle-aged adults. Specifically, age-related changes such as natural loss of muscle mass (45), reduced insulin sensitivity (46) and declined β-cell function (47) significantly weaken the compensatory capacity of older adults to metabolic disorders (48), diluting the marginal effect of metabolic abnormalities on frailty. In contrast, the metabolic system of young and middle-aged adults has not undergone irreversible damage, and metabolic disorders at this stage have a more direct impact on muscle function and energy metabolism; meanwhile, this population has higher compliance with lifestyle interventions (49). Early monitoring and timely intervention of TyG-CVAI may therefore be more effective in delaying the progression of frailty. The prospective association was stronger in ethnic minority participants than in Han participants, which may be explained by the fact that metabolic indicators such as BMI, waist circumference and triglycerides are significantly higher in ethnic minority populations in China than in the Han population (50). In addition, the dietary patterns of some ethnic minorities are dominated by foods high in saturated fatty acids and energy, with insufficient intake of fresh fruits and vegetables. Such pro-inflammatory dietary patterns are prone to induce metabolic syndrome (51, 52), and genetic differences (53) may also exacerbate the risk of metabolic disorders in ethnic minority populations, making TyG-CVAI more closely associated with frailty in these groups.

The prospective association was significantly stronger in participants without hypertension or diabetes than in those with these conditions, suggesting that hypertension and diabetes may be associated with frailty through alternative pathological pathways. Hypertension may be associated with arteriosclerosis and multiple organ damage (52, 54) through hemodynamic overload, while diabetes may damage nerves and blood vessels through glucotoxicity and lipotoxicity (55, 56). These pathological processes are independent of the insulin resistance-visceral fat pathway reflected by TyG-CVAI, leading to a diluted association between TyG-CVAI and frailty in participants with these comorbidities. In participants without underlying chronic diseases, metabolic disorders may represent a more prominent factor associated with frailty, resulting in a stronger observed association. The public health implication of this heterogeneity is that adults under 60 years old, ethnic minorities and individuals without hypertension or diabetes could be prioritized as key target populations for early frailty screening, and TyG-CVAI monitoring could facilitate targeted prevention and control.

Exposure-response association analysis showed a significant non-linear association between baseline TyG-CVAI and follow-up FI, suggesting a potential gradient association. When TyG-CVAI was at a low level, its increase was more closely associated with the elevation of FI, whereas the marginal strength of the association diminished beyond a certain threshold. This pattern is consistent with the compensatory-decompensatory pathological process of metabolic disorders: in the state of mild insulin resistance, pancreatic islet β-cells can maintain blood glucose homeostasis by increasing insulin secretion, during which metabolic disorders are associated with relatively mild bodily changes and leave ample room for intervention (55, 57). With the continuous elevation of TyG-CVAI, the compensatory capacity of β-cells becomes exhausted and enters a decompensatory stage, accompanied by significant increases in blood glucose and lipid levels and subsequent glucotoxic and lipotoxic damage (55–57). At this stage, multi-system functions of the body have undergone irreversible changes, and the incremental association of TyG-CVAI with frailty is weakened. This non-linear relationship provides a clinical implication that interventions targeting TyG-CVAI may be more effective if implemented before the threshold is reached, to maximize the potential reduction of frailty risk. The approximately linear association between baseline FI and follow-up TyG-CVAI further confirmed the weaker magnitude of the reverse path, indicating that the association of frailty with metabolic disorders is mild and sustained without obvious threshold characteristics.

## 5 Limitations

This study has several limitations. First, some FI items were self-reported and may therefore be subject to recall bias; validated standardized questionnaires were used to minimize measurement error. Second, the availability of only one follow-up assessment limited our ability to characterize the long-term dynamic trajectories of TyG-CVAI and FI. Future studies should use multiwave designs and random-intercept cross-lagged panel models (RI-CLPMs) to distinguish within-person from between-person effects and strengthen temporal inference. Third, participants were drawn from a multiethnic cohort in Southwest China. Although this geographic concentration may limit generalizability, it also increased population diversity. Fourth, data on potential confounders, including dietary patterns, physical activity intensity, and medication use, were incomplete and could not be included as independent covariates. Although key covariates were adjusted for, residual confounding may remain. Fifth, the final analytic sample was younger, more highly educated, and had a lower prevalence of hypertension than the overall baseline population. This selection toward healthier participants could plausibly have led to an underestimation of the true association between TyG-CVAI and frailty, potentially rendering our conclusions more conservative. Sixth, as a composite index, TyG-CVAI cannot disentangle the independent contributions of its components. In particular, fasting triglycerides are included in both the TyG index and CVAI, which may introduce mathematical redundancy and partly inflate the apparent advantage of TyG-CVAI over its individual components. However, removing triglycerides from CVAI would fundamentally alter its validated formula and clinical interpretation. Therefore, we instead conducted supplementary cross-lagged analyses using TyG and CVAI separately, in which the composite TyG-CVAI showed better model fit. Future studies should explore alternative composite strategies that minimize component overlap while preserving clinical interpretability. Seventh, some fit indices of the primary model did not reach conventional thresholds for excellent fit. This may reflect known methodological features of two-wave observed-variable cross-lagged models, in which CFI/TLI can be systematically underestimated in simple path models and RMSEA can be overestimated in moderate-to-large samples. Nevertheless, the SRMR (0.097) was close to the acceptable threshold, and the core path coefficients remained highly consistent across all sensitivity analyses, supporting the robustness of the findings.

## 6 Conclusion

Using a prospective cohort design and cross-lagged model analysis, this study identified a significant bidirectional temporal association between TyG-CVAI and FI, with the positive prospective temporal association from TyG-CVAI to FI predominating. This finding suggests that metabolic dysregulation may serve as a temporally preceding risk marker for the development and progression of frailty. However, cross-lagged analysis can establish temporal ordering but cannot confirm causality; therefore, these findings should be regarded as directional evidence requiring further validation. The prospective temporal association between TyG-CVAI and frailty showed significant population heterogeneity and was more pronounced among young and middle-aged adults, ethnic minority populations, and individuals without hypertension or diabetes, thereby providing specific target populations for precision screening of frailty. Constructed based on routine clinical tests in primary care settings, TyG-CVAI is highly standardized, operable and has the potential for large-scale clinical application. It overcomes the limitation of traditional frailty screening that focuses only on older adults, and provides a new strategy for the early prevention and intervention of frailty. The results of this study enrich the longitudinal evidence on the association between metabolic abnormalities and frailty, offer a new perspective for understanding the potential metabolic pathways related to frailty, and provide an important reference for the development of frailty prevention and control strategies adapted to the characteristics of multi-ethnic populations in southwestern China. These findings are exploratory and require further validation in independent cohorts before broader conclusions can be drawn.

## Data Availability

Due to the nature of this research using data from the China Multi-Ethnic Cohort Study (CMEC), the research data is not be made publicly available, further inquiries can be contacted to the corresponding author.
