# Supplementary material for: Cross-lag association between TyG-CVAI and frailty index: evidence from a natural population cohort in southwestern China
Source: Front Public Health. 2026 Sep 14;14:1892429. doi: 10.3389/fpubh.2026.1892429 (PMC13617050; doi:10.3389/fpubh.2026.1892429)
Supplement: Supplementary file 1 [file Data_Sheet_1.docx]

**Supplementary Materials**

**Sup.Table 1 The comparison of the basic characteristics between participants included in this study and the total sample population of CMEC**

| **Variables** | **Overall**  **(N = 99,556)** | **Included**  **(N = 2,476)** | **Not included**  **(N = 97,080)** | ***P* values** | **Missing**  **Number** |
| --- | --- | --- | --- | --- | --- |
| Age (years) | 51.37±11.81 | 48.85±11.66 | 51.43±11.80 | < 0.001 | 0 |
| Age level |  |  |  | < 0.001 | 0 |
| < 60 years old | 73,417 (73.7) | 1,958 (79.1) | 71,459 (73.6) |  |  |
| ≥ 60 years old | 26,139 (26.3) | 518 (20.9) | 25,621 (26.4) |  |  |
| Sex |  |  |  | 0.002 | 0 |
| Male | 59,762 (60.0) | 1,411 (57.0) | 58,351 (60.1) |  |  |
| Female | 39,794 (40.0) | 1,065 (43.0) | 38,729 (39.9) |  |  |
| Ethnicity |  |  |  | < 0.001 | 0 |
| Han | 44,900 (45.1) | 1,772 (71.6) | 43,128 (44.4) |  |  |
| Minority | 54,656 (54.9) | 704 (28.4) | 53,952 (55.6) |  |  |
| Educational level |  |  |  | < 0.001 | 4 |
| < High school degree | 77,403 (77.7) | 1,707 (68.9) | 75,696 (78.0) |  |  |
| ≥ High school degree | 22,149 (22.2) | 769 (31.1) | 21,380 (22.0) |  |  |
| Marital status |  |  |  | 0.026 | 4 |
| Married | 87,901 (88.3) | 2,222 (89.7) | 85,679 (88.3) |  |  |
| Divorced or single | 11,651 (11.7) | 254 (10.3) | 11,397 (11.7) |  |  |
| Smoking history | 20,142 (20.2) | 513 (20.7) | 19,629 (20.2) | 0.562 | 20 |
| Alcohol consumption | 41,813 (42.0) | 1,044 (42.2) | 40,769 (42.0) | 0.894 | 31 |
| Hypertension | 31,879 (32.0) | 655 (26.5) | 31,224 (32.2) | < 0.001 | 13 |
| Chronic kidney diseases | 4,149 (13.6) | 306 (12.4) | 3,843 (13.7) | 0.058 | 69,124 |
| Diabetes | 10,584 (10.6) | 255 (10.3) | 10,329 (10.6) | 0.608 | 13 |

**Sup.Figure 1. The sensitive analysis of the cross-lagged effects path diagram between FI and TyG-CVAI at the baseline and follow-up time points, in participants from the CMEC. The path coefficients all have been standardized, the curved bidirectional arrows represent the covariates considered by the model, the FI was recalculated and the components related to BMI were excluded.**

**Sup.Figure 2. The sensitive analysis of the cross-lagged effects path diagram between FI and TyG-CVAI at the baseline and follow-up time points, in participants from the CMEC. The path coefficients all have been standardized, the curved bidirectional arrows represent the covariates considered by the model, the FI was recalculated and the components related to BMI and diabetes were excluded.**

**Sup.Figure 3. The sensitive analysis of the cross-lagged effects path diagram between FI and TyG-CVAI at the baseline and follow-up time points, in participants from the CMEC. The path coefficients all have been standardized, the curved bidirectional arrows represent the covariates considered by the model, the FI was recalculated and the components related to BMI, diabetes and CKD were excluded.**

**Sup.Figure 4. The sensitive analysis of the cross-lagged effects path diagram between FI and TyG-CVAI at the baseline and follow-up time points, in participants from the CMEC. The path coefficients all have been standardized, the curved bidirectional arrows represent the covariates considered by the model, the FI was recalculated and the components related to BMI, diabetes, CKD and WHR were excluded.**

**Sup.Figure 5. The sensitive analysis of the cross-lagged effects path diagram between FI and TyG at the baseline and follow-up time points, in participants from the CMEC. The path coefficients all have been standardized, and the curved bidirectional arrows represent the covariates considered by the model.**

**Sup.Figure 6. The sensitive analysis of the cross-lagged effects path diagram between FI and CVAI at the baseline and follow-up time points, in participants from the CMEC. The path coefficients all have been standardized, and the curved bidirectional arrows represent the covariates considered by the model.**

**Sup.Table 2 The performance of AIC and BIC for nonlinear-trend models with different numbers of knots**

| **Model** | **Number of knots** | **AIC** | **BIC** |
| --- | --- | --- | --- |
| Baseline FI to follow-up TyG-CVAI | 3 | 34459.91 | 34547.13 |
| Baseline FI to follow-up TyG-CVAI | 4 | 34461.78 | 34554.81 |
| Baseline FI to follow-up TyG-CVAI | 5 | 34463.13 | 34561.98 |
| Baseline FI to follow-up TyG-CVAI | 6 | 34460.98 | 34565.64 |
| Baseline TyG-CVAI to follow-up FI | 3 | 7755.92 | 7843.13 |
| Baseline TyG-CVAI to follow-up FI | 4 | 7757.32 | 7850.35 |
| Baseline TyG-CVAI to follow-up FI | 5 | 7759.20 | 7858.04 |
| Baseline TyG-CVAI to follow-up FI | 6 | 7759.24 | 7863.90 |

Notes: AIC, akaike information criterion; BIC, bayesian information criterion.

**Sup.Table 3 The construction of frailty index and the presentation of its components**

Frailty index (FI) was constructed based on 25 health deficit items as listed in Sup.Table 2. For each participant, the FI score was calculated as the ratio of the number of deficits present to the total number of deficits considered. According to the calculated FI scores, participants were categorized into three frailty states: robust (FI ≤ 0.1), prefrail (0.1 < FI < 0.25), and frail (FI ≥ 0.25).

| No. | Definition according to baseline self-report and/or measurement | Coding of variables |
| --- | --- | --- |
| 1 | Self-reported doctor diagnosis of hypertension,  self-reported use of antihypertension drugs,  measured SBP ≥140 mmHg, or measured DBP ≥90 mmHg | Yes = 1, No = 0 |
| 2 | Self-reported doctor diagnosis of heart disease | Yes = 1, No = 0 |
| 3 | Self-reported doctor diagnosis of stroke or transient ischemic attack | Yes = 1, No = 0 |
| 4 | Self-reported doctor diagnosis of emphysema or chronic bronchitis | Yes = 1, No = 0 |
| 5 | Self-reported doctor diagnosis of tuberculosis | Yes = 1, No = 0 |
| 6 | Self-reported doctor diagnosis of asthma | Yes = 1, No = 0 |
| 7 | Self-reported doctor diagnosis of peptic ulcer | Yes = 1, No = 0 |
| 8 | Self-reported doctor diagnosis of gallstone disease | Yes = 1, No = 0 |
| 9 | Self-reported doctor diagnosis of rheumatoid arthritis | Yes = 1, No = 0 |
| 10 | Self-reported doctor diagnosis of fracture | Yes = 1, No = 0 |
| 11 | Self-reported doctor diagnosis of neurasthenia | Yes = 1, No = 0 |
| 12 | Self-reported doctor diagnosis of diabetes,  measured fasting blood glucose ≥7.0 mmol/L,  or measured random blood glucose ≥11.1 mmol/L | Yes = 1, No = 0 |
| 13 | Self-reported doctor diagnosis of cancer | Yes = 1, No = 0 |
| 14 | Self-reported doctor diagnosis of chronic kidney disease | Yes = 1, No = 0 |
| 15 | During the past month, did you have any of the following items:  (1) taking >30 minutes to fall asleep after going bed or  waking up in the middle of the night (≥3days/week);  (2) waking up early and not being able to go back to sleep(≥3days/week);  (3) taking sleeping pills to help sleep (≥1day/week); or  (4) having difficulty staying alert while at work, eating  or meeting people during daytime (≥3days/week)? | Yes = 1, No = 0 |
| 16 | How often do you have bowel movements each week? | Less than 3 times a week = 1,  Others = 0 |
| 17 | Do your gums often bleed when you brush your teeth? | Yes = 1, No = 0 |
| 18 | Total physical activity | <20th percentile (sex-specified) = 1,  Others = 0 |
| 19 | During the past 12 months, have you lost weight ≥2.5kg  and not tried to reduce weight intentionally? | Yes = 1, No = 0 |
| 20 | During the past 12 months, did you feel much more sad,  or depressed than usual for two or more weeks? | Yes = 1, No = 0 |
| 21 | How is your current general health status? | Very poor = 1, Poor = 0.75,  Fair = 0.5, Good = 0.25,  Excellent = 0 |
| 22 | Body mass index | ≥28 = 1, 24 - 28 = 0.5, <28 = 0 |
| 23 | Waist-to-hip ratio | For male:  ≥0.95 = 1, 0.90-0.95 = 0.5, <0.90 = 0; For female:  ≥0.90 = 1, 0.85-0.90 = 0.5, <0.85 = 0; |
| 24 | Measured heart rate | <60 or >100 = 1, Others = 0 |
| 25 | Peak expiratory flow | <450L/min or >600L/min = 1, Others = 0 |
